# Supplementary material for: The Association between Metabolically Healthy Obesity, Cardiovascular Disease, and All-Cause Mortality Risk in Asia: A Systematic Review and Meta-Analysis
Source: Int J Environ Res Public Health. 2020 Feb 19;17(4):1320. doi: 10.3390/ijerph17041320 (PMC7068615; doi:10.3390/ijerph17041320)
Supplement: Supplementary file 1 [file ijerph-17-01320-s001.pdf]

Supplementary Materials

Table S1. PRISMA checklist.

Section/topic	#	Checklist item	Reported on page #
TITLE			
Title	1	Identify the report as a systematic review, meta-analysis, or both.	1
ABSTRACT			
Structured summary	2	Provide a structured summary including, as applicable: background; objectives; data sources; study eligibility criteria, participants, and interventions; study appraisal and synthesis methods; results; limitations; conclusions and implications of key findings; systematic review registration number.	2
INTRODUCTION			
Rationale	3	Describe the rationale for the review in the context of what is already known.	2
Objectives	4	Provide an explicit statement of questions being addressed with reference to participants, interventions, comparisons, outcomes, and study design (PICOS).	3
METHODS			
Protocol and registration	5	Indicate if a review protocol exists, if and where it can be accessed (e.g., Web address), and, if available, provide registration information including registration number.	3
Eligibility criteria	6	Specify study characteristics (e.g., PICOS, length of follow-up) and report characteristics (e.g., years considered, language, publication status) used as criteria for eligibility, giving rationale.	3
Information sources	7	Describe all information sources (e.g., databases with dates of coverage, contact with study authors to identify additional studies) in the search and date last searched.	3
Search	8	Present full electronic search strategy for at least one database, including any limits used, such that it could be repeated.	3;Supplement 3-9

Study selection	9	State the process for selecting studies (i.e., screening, eligibility, included in systematic review, and, if applicable, included in the meta-analysis).	3
Data collection process	10	Describe method of data extraction from reports (e.g., piloted forms, independently, in duplicate) and any processes for obtaining and confirming data from investigators.	4
Data items	11	List and define all variables for which data were sought (e.g., PICOS, funding sources) and any assumptions and simplifications made.	3-4
Risk of bias in individual studies	12	Describe methods used for assessing risk of bias of individual studies (including specification of whether this was done at the study or outcome level), and how this information is to be used in any data synthesis.	4
Summary measures	13	State the principal summary measures (e.g., risk ratio, difference in means).	4
Synthesis of results	14	Describe the methods of handling data and combining results of studies, if done, including measures of consistency (e.g., I^2) for each meta-analysis.	4
Section/topic	#	Checklist item	Reported on page #
Risk of bias across studies	15	Specify any assessment of risk of bias that may affect the cumulative evidence (e.g., publication bias, selective reporting within studies).	4
Additional analyses	16	Describe methods of additional analyses (e.g., sensitivity or subgroup analyses, meta-regression), if done, indicating which were pre-specified.	4
RESULTS			
Study selection	17	Give numbers of studies screened, assessed for eligibility, and included in the review, with reasons for exclusions at each stage, ideally with a flow diagram.	5
Study characteristics	18	For each study, present characteristics for which data were extracted (e.g., study size, PICOS, follow-up period) and provide the citations.	Supplement 9-16
Risk of bias within studies	19	Present data on risk of bias of each study and, if available, any outcome level assessment (see item 12).	Supplement 17-18

Results of individual studies	20	For all outcomes considered (benefits or harms), present, for each study: (a) simple summary data for each intervention group (b) effect estimates and confidence intervals, ideally with a forest plot.	Supplement 9-16
Synthesis of results	21	Present results of each meta-analysis done, including confidence intervals and measures of consistency.	6-8
Risk of bias across studies	22	Present results of any assessment of risk of bias across studies (see Item 15).	9
Additional analysis	23	Give results of additional analyses, if done (e.g., sensitivity or subgroup analyses, meta-regression [see Item 16]).	8
DISCUSSION			
Summary of evidence	24	Summarize the main findings including the strength of evidence for each main outcome; consider their relevance to key groups (e.g., healthcare providers, users, and policy makers).	9
Limitations	25	Discuss limitations at study and outcome level (e.g., risk of bias), and at review-level (e.g., incomplete retrieval of identified research, reporting bias).	10
Conclusions	26	Provide a general interpretation of the results in the context of other evidence, and implications for future research.	10
FUNDING			
Funding	27	Describe sources of funding for the systematic review and other support (e.g., supply of data); role of funders for the systematic review.	11

Table S2. Search strategy.

Search strategy in Pubmed

PICO	Search	Query
**PIO**	#55	Search (((((((((Obesity, Metabolically Benign) OR Metabolically Benign Obesity) OR Metabolically Healthy Obesity) OR MHO[Title/Abstract] OR obesity paradox OR obesity phenotypes OR obesity phenotype*) OR ((((((((((((((Overweight) OR obesity) OR obese[Title/Abstract]) OR Body Mass Index) OR BMI[Title/Abstract]) OR Quetelet Index[Title/Abstract]) OR fat[Title/Abstract]) OR Body Weight) OR Body Composition) OR fatness[Title/Abstract]) OR body mass[Title/Abstract]) OR Anthropometry) OR adiposity)) AND ((normal[Text Word] OR healthy[Text Word] OR benign[Text Word] OR without[Text Word] OR absence[Text Word]))) AND metabolic))) AND (((((((((((Carotid Artery Diseases OR Cardiovascular Diseases) OR myocardial infarction) OR coronary[Text Word]) OR heart failure) OR Cardiac Disease) OR angina pectoris[Text Word]) OR Vascular Diseases) OR Cardiovascular[Text Word]) OR CVD[Title/Abstract]) OR heart diseases OR cerebrovascular disease) OR stroke[Text Word]) OR All cause[Title/Abstract])) AND (((((((Mortality) OR mortalit*[Title/Abstract]) OR Morbidity) OR Morbidities[Text Word]) OR Incidence) OR incident[Title/Abstract]) OR death[Title/Abstract]))))
**O**	#47	Search ((((((Mortality) OR mortalit*[Title/Abstract]) OR Morbidity) OR Morbidities[Text Word]) OR Incidence) OR incident[Title/Abstract]) OR death[Title/Abstract]
	#46	Search death[Title/Abstract]
	#45	Search incident[Title/Abstract]
	#44	Search Incidence
	#43	Search Morbidities[Text Word]
	#42	Search Morbidity
	#41	Search mortalit*[Title/Abstract]
	#40	Search Mortality
**I**	#39	Search (((((((((((Cardiovascular Diseases) OR myocardial infarction) OR coronary[Text Word]) OR heart failure) OR Cardiac Disease) OR angina pectoris[Text Word]) OR Vascular Diseases) OR Cardiovascular[Text Word]) OR CVD[Title/Abstract]) OR heart diseases) OR stroke[Text Word]) OR All cause[Title/Abstract]
	#56	Search cerebrovascular disease
	#53	Search Carotid Artery Diseases Sort by: [pubsolr12]

	#38	Search All cause[Title/Abstract]
	#37	Search stroke[Text Word]
	#36	Search heart diseases
	#35	Search CVD[Title/Abstract]
	#34	Search Cardiovascular[Text Word]
	#33	Search Vascular Diseases
	#32	Search angina pectoris[Text Word]
	#31	Search Cardiac Disease
	#30	Search heart failure
	#29	Search coronary[Text Word]
	#28	Search myocardial infarction
	#26	Search Cardiovascular Diseases
**P**	#25	Search ((((Obesity, Metabolically Benign) OR Metabolically Benign Obesity) OR Metabolically Healthy Obesity) OR MHO[Title/Abstract] OR obesity paradox OR obesity phenotypes OR obesity phenotype*) OR ((((((((((((((Overweight) OR obesity) OR obese[Title/Abstract]) OR Body Mass Index) OR BMI[Title/Abstract]) OR Quetelet Index[Title/Abstract]) OR fat[Title/Abstract]) OR Body Weight) OR Body Composition) OR fatness[Title/Abstract]) OR body mass[Title/Abstract]) OR Anthropometry) OR adiposity)) AND ((normal[Text Word] OR healthy[Text Word] OR benign[Text Word] OR without[Text Word] OR absence[Text Word]))) AND metabolic)
	#24	Search ((((((((((((((Overweight) OR obesity) OR obese[Title/Abstract]) OR Body Mass Index) OR BMI[Title/Abstract]) OR Quetelet Index[Title/Abstract]) OR fat[Title/Abstract]) OR Body Weight) OR Body Composition) OR fatness[Title/Abstract]) OR body mass[Title/Abstract]) OR Anthropometry) OR adiposity)) AND ((normal[Text Word] OR healthy[Text Word] OR benign[Text Word] OR without[Text Word] OR absence[Text Word]))) AND metabolic
	#23	Search metabolic
	#22	Search (normal[Text Word] OR healthy[Text Word] OR benign[Text Word] OR without[Text Word] OR absence[Text Word])

	#19	Search ((((((((((((Overweight) OR obesity) OR obese[Title/Abstract]) OR Body Mass Index) OR BMI[Title/Abstract]) OR Quetelet Index[Title/Abstract]) OR fat[Title/Abstract]) OR Body Weight) OR Body Composition) OR fatness[Title/Abstract]) OR body mass[Title/Abstract]) OR Anthropometry) OR adiposity
	#18	Search adiposity
	#17	Search Anthropometry
	#16	Search body mass[Title/Abstract]
	#15	Search fatness[Title/Abstract]
	#14	Search Body Composition
	#13	Search Body Weight
	#12	Search fat[Title/Abstract]
	#11	Search Quetelet Index[Title/Abstract]
	#10	Search BMI[Title/Abstract]
	#9	Search Body Mass Index
	#8	Search obese[Title/Abstract]
	#7	Search obesity
	#6	Search Overweight
	#5	obesity paradox OR obesity phenotypes OR obesity phenotype*
	#4	Search MHO[Title/Abstract]
	#3	Search Metabolically Healthy Obesity
	#2	Search Metabolically Benign Obesity
	#1	Search Obesity, Metabolically Benign

Search strategy in Embase

PICO	Search	Query
	#41	#38 AND ('Article'/it OR 'Article in Press'/it OR 'Conference Paper'/it OR 'Conference Review'/it OR 'Review'/it OR 'Short Survey'/it)
	#40	#38 AND 'Conference Abstract'/it

PICO	#39	#20 AND #30 AND #38
O	#38	#30 OR #31 OR #32 OR#33 OR #34 OR #35 OR #36
	#37	incidence:ti,ab OR incident:ti,ab
	#36	'incidence'/exp
	#35	morbidities:ti,ab
	#34	'morbidity'/exp
	#33	mortalit*:ti,ab
	#32	'death':ab,ti
	#31	'mortality'/exp
I	#30	#28 OR #29
	#29	all NEXT/2 cause
	#28	#21 OR #22 OR #23 OR #24 OR #25 OR #26 OR #27
	#27	'cardiovascular':ab,ti OR 'myocardial':ab,ti OR 'coronary':ab,ti OR 'heart':ab,ti OR 'angina pectoris':ab,ti OR 'stroke':ab,ti OR 'cvd':ab,ti OR 'cardiac':ab,ti OR 'vascular':ab,ti OR 'cerebrovascular':ab,ti
	#26	'carotid artery disease'/exp
	#25	'cerebrovascular accident'/exp
	#24	'vascular disease'/exp
	#23	'myocardial disease'/exp
	#22	'heart disease'/exp
	#21	'cardiovascular disease'/exp
P	#20	#1 OR #2 OR #3 OR #4 OR #5 OR #6 OR #19
	#19	#6 AND #16 AND #17
	#18	'normal':ti,ab,kw OR 'healthy':ti,ab,kw OR 'benign':ti,ab,kw OR 'without':ti,ab,kw OR 'absence':ti,ab,kw
	#17	#7 OR #8 OR #9 OR #10 OR #11 OR #12 OR #13 OR #14 OR #15 OR #16
	#16	'anthropometric':ti,ab,kw OR 'adiposity':ti,ab,kw
	#15	'anthropometry'/exp

	#14	'body composition'/exp
	#13	'body weight'/exp
	#12	'bmi':ti,ab,kw OR 'body mass index':ti,ab,kw OR 'body mass':ti,ab,kw
	#11	'body mass'/exp
	#10	'obesity':ti,ab,kw
	#9	'adiposit*':ti,ab,kw OR 'obesitas':ti,ab,kw OR 'overweight':ti,ab,kw OR 'obese':ti,ab,kw OR 'fat':ti,ab,kw
	#8	'obesity'/exp
	#7	metaboli*:ti,ab
	#6	'obesity phenotypes' OR (('obesity'/exp OR obesity) AND phenotypes)
	#5	obesity NEAR/3 paradox
	#4	obesity paradox'/exp
	#3	mho:ti,ab
	#2	'metabolically healthy obesity'/exp
	#1	'metabolically benign obesity'/exp

Search strategy in Cochrane Library

PICO	Search	Query
	#1	MeSH descriptor: [Obesity, Metabolically Benign] explode all trees
	#2	(Metabolically Healthy Obesity):ti,ab,kw OR (Metabolically Benign Obesity):ti,ab,kw OR (MHO):ti,ab,kw OR (obesity paradox):ti,ab,kw OR obesity paradox OR obesity phenotypes OR obesity phenotype* (Word variations have been searched)
	#3	#1 OR #2
	#4	(Overweight OR obesity OR obese OR Body Mass Index OR BMI OR Quetelet Index OR fat OR Body Weight OR Body Composition OR fatness OR body mass OR Anthropometry OR adiposity):ti,ab,kw (Word variations have been searched)
	#5	(metaboli*):ti,ab,kw (Word variations have been searched)
	#6	(normal OR healthy OR benign OR without OR absence):ti,ab,kw (Word variations have been searched)
	#7	#4 AND #5 AND #6

**P**	#8	#3 OR #7
	#9	MeSH descriptor: [Cardiovascular Diseases] explode all trees
	#10	MeSH descriptor: [Heart Diseases] explode all trees
	#11	MeSH descriptor: [Vascular Diseases] explode all trees
	#12	MeSH descriptor: [Myocardial Infarction] explode all trees
	#13	(cardiovascular OR myocardial OR coronary OR stroke OR CVD):ti,ab,kw (Word variations have been searched)
	#14	(Heart OR Cardiac OR Vascular OR Cerebrovascular):ti,ab,kw (Word variations have been searched)
	#15	(all cause):ti,ab,kw (Word variations have been searched)
**I**	#16	#9 OR #10 OR #11 OR #12 OR #13 OR #14 OR #15
	#17	MeSH descriptor: [Mortality] explode all trees
	#18	MeSH descriptor: [Morbidity] explode all trees
	#19	MeSH descriptor: [Incidence] explode all trees
	#20	(Morbidity OR Morbidities OR Mortality OR mortalities OR Death Rate OR Incidence):ti,ab,kw (Word variations have been searched)
**O**	#21	#17 OR #18 OR #19 OR #20
**PIO**	**#22**	**#8 AND #16 AND #21**

Table S3. Characteristics of included cohort studies.

Study	Participants	Definition of obesity, BMI categories (kg/m^2)	Definition of metabolic healthy / unhealthy	Adjusted variables	Diagnostic criteria and main results presented by MHO/MHOW compared with MHNW/MHNO with OR (95%CI)	NOS
Hosseinpanah, et al 2011[1]	Iran, TLGS, N=6,215, 56.9 % women, 6.6 % MHO, 44.6± 0.5 y/o in MHO, 14.2% smoker, 8.4 yrs f/u	Measured; NW: 18.5-24.9; OW: 25.0-29.9; OB: ≥30	IDF (cut-point WC ≥89 cm for men, ≥91 cm for women)	Age, sex, PA, smoking, family history of premature CHD, high TC	CVD defined as any CHD events (included MI, angiographically proved CHD, CHD death), stroke (a new neurological deficit lasted ≥24 h) or CVD death<br>Compared with MHNW,<br>fatal and non-fatal CVD of MHOW: 1.10 (0.76-1.61), MHO: 1.07 (0.59-1.96)	9
Choi, et al 2013[2]	Korea, SWS study, N=2,317, 77.9 % women, 25.8% MHO, 69.8 ± 5.6 y/o, 10.9% smoker, 10.3 yrs f/u	Measured; NW: 18.5-23; OW: 23-25; OB: ≥25	Modified ATP-III criteria (without WC criterion; using 2 hPG≥7. 8 mmol/l or treatment for DM instead of FBG≥5. 6 mmol/L)	Age, sex, smoking, alcohol, presence of DM, HTN, CVD	Death certificate data from Korean National Statistical Office, CV death by ICD-10 codes<br>Compared with MHOW,<br>CVD of MHO: 1.78 (0.94-3.4)<br>All-cause mortality of MHO: 1.18 (0.84-1.68)	8
Keihani, et al 2015[3]	Iran, TLGS, N=6,430, 57.3% women, 47.4 ±12.4 y/o, 12.4% MHO, 14.6%	Measured WC ≥89 cm for men, ≥91 cm for	Modified JIS criteria (except WC)<br>MH if ≤1 criteria	Age, sex, smoking, educational level, PA, and history of premature CAD in family, FBS, TG, HDL-C,	CVD defined as any CHD events (MI, angiographically proved CHD, CHD death), stroke (a new neurological deficit lasted ≥24 h) or CVD death. Compared with MHNO, CVD of MHO: 1.64 (1.09-2.47)	9

	smoker, 15.3% smoker in MHAO, 10 yrs f/u	women	N=3844. Insulin sensitive: HOMA-IR < 2.5 (mole×μU/L^2); insulin resistant: HOMA-IR≥2.5 (mole×μU/L^2)	SBP	Compared with MHNO<br>CVD of MHO: 2.58 (1.21-5.49)	
Luo, et al 2015[4]	China, SHDS, N=2,764, 57.1% women, 30-90 y/o, 50.0 y/o in MHO, 24.8% MHO, 3.7 yrs f/u	BF>25% for men; BF>35% for women	JCDCG criteria<br>MH if ≤2 criteria	Age, TC, TG, family history of DM and CVD	CVD events defined as the first occurrence of CHD or stroke; stroke defined as ICH or cerebral infarction. Compared with MHNO,<br>CVD in MHO men: 0.98 (0.51-1.89)<br>CVD in MHO women: 1.10 (0.59-2.05)<br>Stroke in MHO men: 1.27 (0.62-2.61)<br>Stroke in MHO women: 2.21 (0.94-5.17)<br>CHD in MHO men: 0.43 (0.1-1.93)<br>CHD in MHO women: 0.53 (0.19-1.44)	6
Mirbolouk, et al 2015[5]	Iran, TLGS, N= 1,199, 41.8% women,<br>18.8% MHO,<br>11.7% smoker,<br>70.0 ± 4.6 years,<br>9.74 yrs f/u	Measured; NW: <25; OW:25-29.9; OB: ≥30	JIS criteria	Age, sex, smoking, TC and lipid-lowering drugs	CVD defined as any CHD events (MI, angiographic proven CHD), stroke (a new neurological deficit lasted >24 h) or CVD death. Compared with MHOW,<br>CVD events of MHOB:1.46 (0.64-3.34)<br>CVD mortality of MHO: 1.07 (0.13-8.78)<br>All-cause mortality of MHO: 1.33 (0.51; 3.47)	8
Sung, et al 2015[6]	Korea, N=275,867,<br>43.4 % women, 6.7% MHO, 40.2 y/o, 72.8% smoker, 8 yrs f/u	Measured; NW/NO: 18.5-24.9; OB: ≥25	JIS<br>MH if none of the criteria	All-cause mortality: age, sex, smoking, alcohol, PA, education, DM, HTN, history of COPD, CKD, stroke, liver disease, hepatitis;	Causes of death by ICD-10<br>Compared with MHNO,<br>CV mortality of MHO: 0.42 (0.10-1.79)<br>All-cause mortality of MHO: 0.71 (0.46-1.09)	8

				CV mortality: age, sex, smoking, alcohol, PA, education, HTN, history of CVD		
Twig, et al 2015[7]	Israel, MELANY study, N= 31,684, 0% women, 1.9% MHO, 31.4 ± 5.7 y/o, 28% smoker, 6.1 yrs f/u	Measured; NW: 18.5-24.9; OW:25-29.9; OB: ≥30	ATP-III criteria	Age, family history of CAD, LDL-C, WBC count, smoking, PA	Based on diagnostic procedure treadmill exercise test, ST segment depression, symptoms of angina, coronary angiography<br>Compared with MHNW,<br>CHD of MHOW: 1.86(0.77-4.49); MHO: 5.08 (1.69-11.24)	8
Kim, et al 2016[8]	Korea, KoGES , N=7,588, 15.5% MHO, 58.2 % women in MHO, 49.9 y/o in MHO, 39.6% smoker, 8.2±2.7 yrs f/u	Measured NO: <25; OB: ≥25	Modified ATP-III criteria (except WC criteria)<br>MH if ≤1 criteria	Age, sex, study site, PA, smoking, alcohol	Fatal CVD: ICD-10 (I00–I79). Nonfatal CVD: MI, CHD, CHF, stroke (determined by questionnaire as newly developed CV events)<br>Compared with MHNO,<br>baseline MHO: 1.4 (0.99-1.8)<br>persistent MHO: 2.1 (1.2–3.7)	8
Yang, et al 2016[9]	Korea, N=323,175, 16.5% MHO, 37.5 % women in MHO, 29.7% smoker, ≥20 y/o, 8 yrs f/u	Record from database NW/NO: 18.5-<25; OB: ≥25	MH if none of the following: 1)DM: medication under ICD-10 or FBG ≥7 mmol/L; 2) HTN: medication under ICD-10 or BP≥140/90 mmHg; 3) Dyslipidemia: medication under ICD-10 or TC ≥6.21 mmol/L	Age, sex, smoking, alcohol, PA, income	Cause of death classified by ICD-10<br>Compared with MHNO,<br>CV mortality of MHO: 0.73 (0.57-0.95)<br>All-cause mortality of MHO: 0.81 (0.74-0.88)	8

Doustmohamadian, et al 2017[10]	Iran, TLGS study, N=8,804, 54.8% women, 12.8% MHO, 47.7 y/o, 15% smoker, 12 yrs f/u	Measured WC≥89 cm for men; WC≥91 cm for women	JIS criteria<br>MH if ≦2 of the criteria	Age, sex, smoking, education, PA	Not mentioned<br>Compared with MHNW,<br>All-cause mortality of MHO: 1.35 (0.89; 2.03)	7
Fujihara, et al 2017[11]	Japan,<br>N= 123,746,<br>0% women,<br>7.3 % MHO,<br>≥ 18 y/o, 42.7% smoker, 4.1 yrs f/u	Measured; NO: <25; OB: ≥25	MH if <1 of the following :<br>1)BP≥135/85 mmHg or medication; 2)TG ≥1.7 mmol/L or medication; 3)HDL-C < 1.03 mmol/L (men), <1.29 mmol/L (women)	Age, BMI, smoking, LDL-C level	Cardiac fatal and non-fatal events (excluding HF) and for medical procedures<br>Compared with MHNO with NGT, CVD of MHO with NGT:1.18 (0.49-2.84)<br>Compared with MHNO with prediabetes, CVD of MHO with prediabetes : 0.84 (0.30-2.39)<br>Compared with MHNO with diabetes, CVD of MHO with diabetes: 4.15 (1.73-9.98)	7
Mirzaei, et al 2017[12]	Iran, TLGS, N=7,842, 55.2% women, 2.0% MHO, 41.8 y/o in MHO, 15.3% smoker,11.9 yrs f/u	Measured; NW: 18.5-24.9; OW: 25.0-29.9; OB: ≥30	JIS criteria<br>Insulin sensitive: HOMA-IR < 2.6 (mole×μU/L^2); Insulin resistant: HOMA-IR≥2.6 (mole×μU/L^2)	Age, sex, smoking, education, PA, family history of premature CHD, TC	CVD events: defined as any CHD, stroke (a new neurological deficit that lasted ≥24 h), or CVD death (fatal CHD or fatal stroke)<br>Compared with MHNW, CVD of MHOW: 1.22 (0.73-2.04); MHO: 1.74 (0.68-4.44)<br>Compared with MHNW, CVD of MHOW: 1.70 (1.13-2.55); MHO: 1.96 (1.18; 3.24)	9
Lee, et al 2018[13]	Korea, NHIS-NSC, N= 354,083, 47.3 % women,<br>7.5 % MHO<br>41.7 y/o in MHO,<br>7.4 yrs f/u	Measured; NW/NO: 18.5-24.9; OB: ≥25	Modified JIS, MH if <1 of the following:<br>1)BP≥130/85 mmHg or medication; 2)FBG ≥100 mg/dL or medication; 3)TC≥240 mg/dL or	Age, sex, income, area, smoking, drinking, exercise, history of IHD, PAOD, HF, TIA, venous thromboembolism, COPD, ESRD, liver	Ischemic stroke by ICD 10 codes combined with images<br>Compared with MHNO, stoke of MHO: 0.99 (0.81-1.20)	8

			medication	cirrhosis, cancer, cardiac surgery		
Li, et al 2018[14]	China, Beijing cohort study, N=9,393, 65.9% women, 6.7% MHO, 16.6% smoker in MHO, 56.5 y/o, 3.2 yrs f/u	Measured; NW: 18.5-<24; OW: 24.0–27.9; OB: ≥28	ATP-III criteria	Sex, age, income, education, PA, smoking, drinking, ideal diet, family history of CVD, LDL-C	CVD (admission for MI, coronary revascularization, HF or stroke) using epidemiological questionnaire<br>Compared with MHNW,<br>CVD events of MHOW: 1.09(0.7;1.7); MHO: 1.91(1.13; 3.24)	6
Xu, et al 2018[15]	China, Kailuan study, N=91,866, 7.3% MHO, 19.9% women in MHO, 33.7% smoker, 18-98 y/o, 8 yrs f/u	Measured; NW: 18.5-23.9; OW: 24.0–27.9; OB: ≥28	IDF criteria	Age, sex, education, income, smoking, drinking, PA, sodium intake, LDL-C, hs-CRP, eGFR	MI and death due to MI, identified from medical records and death certificates<br>Compared with MHNW, MI of MHOW: 1.08(0.89;1.31); MHO: 1.76(1.37; 2.25)	9
Zhang, et al 2018[16]	China, N=3,485, 0% women,<br>7% MHO, >60 y/o, 15.9% smoker, 5 yrs f/u	Measured; NW: <24; OW: 24.0–27.9; OB: ≥28	MH if none of the following criteria: 1) HTN; 2) DM; 3) dyslipidemia	Age, smoking, SBP, FBS, TC, LDL-C, Cr	Mortality defined by ICD-10<br>Compared with MHNW,<br>all-cause mortality of MHOW: 0.86(0.55;1.35); MHO: 1.56 (0.85; 2.86)<br>CV mortality of MHOW:0.96(0.51;1.81); MHO: 1.40 (0.56; 3.51)	8
Cho, et al 2019[17]	Korea, NHIS-HEALS<br>N= 362,863, 46.3 % women,<br>10.0 % MHO	Measured; NO: <25; OB: ≥25	Modified ATP-III criteria (except WC criteria)<br>MH if ≤1 of the criteria	Age, sex, smoking, alcohol, PA, LDL-C l	Cause of death according to ICD-10 codes<br>CV events were defined as admissions for MI and stroke according to ICD-10<br>Compared with MHNO,<br>CVD of MHO: 1.14 (1.05-1.24)	7

	57.4 y/o in MHO, 35.7% smoker, 4 yrs f/u				CVD mortality of MHO: 0.85 (0.69-1.06)<br>all-cause mortality of MHO: 0.86 (0.79-0.93)	
Li, et al 2019[18]	China, CHRLS, N=7,849, 52.8 % women, 10.1% MHO 55.3 y/o in MHO, 30.8% smoker, 3.6 yrs f/u	Measured; NW: 18.5-23.9; OW/OB: ≥24	MH if ≤1 of the following: 1)BP≥130/85mmHg or diagnosed or medication; 2)FBG ≥100 mg/dL or HbA1c ≥6.0% or diagnosed or medication; 3)TG ≥150 mg/dL or medication; 4)HDL-C < 40 mg/dL (men), <50 mg/dL (women); 5) DM; 6)hs-CRP ≥3 mg/L	Age, sex, residence, educational, marital status, smoking, alcohol, PA, history of arthritis, asthma, lung disease and fall, physical impairments in ADL, IADL, cognitive score, TC, HDL-C	CVD by self-reported doctor's diagnosis of heart diseases (heart attack, CHD, angina, CHF or other heart problems) and stroke.<br>Compared with MHNO,<br>CVD of MHO: 1.33 (1.19-1.49)	6
Izumida, et al 2019[19]	Japan, JMS, N=10,824, 61% women, 0.6% MHO, 55.3 y/o, 36 % smoker, 18.4 yrs f/u	Measured; NW: 18.5–24.9; OW: 25.0–29.9; OB: ≥30.0	Modified ATP-III criteria (except WC criteria) MH if ≤2 of the criteria	Age, sex, TC, smoking, drinking status, education, marital status, PA, sleeping hours	Mortality based on the Cause-of-Death Registry according to ICD-10 codes<br>Compared with MHNW,<br>Aged <65 Years,<br>CVD death of MHO: 1.0 (0.1–7.2)<br>all-cause mortality of MHO: 1.3 (0.6–2.9)<br>Aged ≥65 Years,<br>CVD death of MHO: 0.9 (0.1–6.5)<br>all-cause mortality of MHO: 1.0 (0.4–2.7)	9

Abbreviations: 2hPG, 2 h plasma glucose; ADL, activities of daily living; BF, body fat; BMI, body mass index; BP, blood pressure; CABG, coronary artery bypass graft; CAD, coronary artery disease; CHD, coronary heart disease; CHF, congestive heart failure; CHRLS, China Health and Retirement Longitudinal Study; CI, confidence interval; CKD, chronic kidney disease; COPD, chronic obstructive pulmonary disease; CV, cardiovascular; CVD, cardiovascular disease; DM, Diabetes

mellitus; ESRD, end stage renal disease; FBG, fasting blood glucose; FPG, fasting plasma glucose; f/u, follow up; HDL-C, high-density lipoprotein cholesterol; HF, heart failure; HOMA-IR, homeostasis model assessment-insulin resistance; HR, hazard ratio; hs-CRP, high sensitivity C-reactive protein; HTN, hypertension; IADL, Independent activities of daily living; ICD, International Classification of Disease; ICH, intracranial hemorrhage; IDF, International Diabetes Federation; IHD, ischemic heart disease; IS-NAO, insulin sensitive non-abdominal obese; IS-AO, insulin sensitive abdominal obese; IR-NAO, insulin resistant non-abdominal obese; IR-AO, insulin resistant abdominal obese; IRR, Incidence rate ratio; JIS, Joint Interim Statement; JCDCG, Joint Committee for Developing Chinese Guidelines; JMS, Jichi Medical School; KAMIR-NIH, Korea Acute Myocardial Infarction Registry National Institutes of Health registry; KoGES, Korean Genome and Epidemiology Study; L, liter; LDL-C, low-density lipoprotein cholesterol; LVEF, left ventricular ejection fraction; MACE, Major adverse cardiovascular event; MELANY cohort, Metabolic, Lifestyle and Nutrition Assessment in Young Adult Cohort; MetS, Metabolic syndrome; MH, metabolically healthy; MI, MHAO, metabolically healthy abdominal obese; MHNAO, metabolically healthy non-abdominal obese; myocardial infarction; MHNO, metabolically healthy non-obese; MHNW, metabolically healthy normal weight; MHO, metabolically healthy obesity; MHOW, metabolically healthy overweight; NCEP-ATP III, National Cholesterol Education Program- Adult Treatment program III;NGT, normal glucose tolerance; NHIS-HEALS, National Health Insurance Service-National Health Screening Cohort; NW, normal weight; OB, obese; OW, overweight; PA, physical activity; PAOD, peripheral artery occlusion disease; PCI, percutaneous coronary intervention; SBP, systolic blood pressure; SHDS, Shanghai Diabetes Study; SWS study, South-West Seoul Study; TC, total cholesterol; TG, triglycerides; TIA, transient ischemic attack; TLGS, Tehran Lipid and Glucose Study; WBC, white blood cell; WC, waist circumstance; y/o, years old; yrs, years

Table S4. Newcastle−Ottawa scale for assessment of quality of included studies – Cohort studies (each star represents if individual criterion within the subsection was fulfilled)

Study	Selection				Comparability		Outcome			**Total quality score**
Quality assessment criteria	Representativeness of exposed cohort	Selection of non−exposed cohort	Ascertainment of exposure	Demonstration that outcome of interest was not present at start of study	Adjust for the most important risk factors	Adjust for other risk factors	Assessment of outcome	Follow−up length	Loss to follow−up rate	
Acceptable (★)	Representative of general adult population in community (age/sex/being at risk of disease)	Drawn from the same community as exposed cohort	Secure records, Structured interview	Yes, or excluded when analysis	Yes, at least for age and sex	Yes, and smoking must be included	Independent blind assessment, record linkage	Follow−up >5 years	Follow−up completed, or small subjects lost (<20%), or lost subjects unlikely to introduce bias*	
Hosseinpanah, et al 2011[1]	★	★	★	★	★	★	★	★	★	**9**
Choi, et al 2013[2]	★	★	★	★	★	★	★	★	−	**8**
Keihani, et al 2015[3]	★	★	★	★	★	★	★	★	★	**9**

Luo, 2015[4]	★	★	★	★	★	-	★	-	-	**6**
Mirbolouk, et al 2015[5]	★	★	★	−	★	★	★	★	★	**8**
Sung, et al 2015[6]	★	★	★	★	★	★	★	★	-	**8**
Twig, et al 2015[7]	★	★	★	★	★	★	★	★	−	**8**
Kim, et al 2016[8]	★	★	★	★	★	★	−	★	★	**8**
Yang, et al 2016[9]	★	★	★	★	★	★	★	★	-	**8**
Doustmohamadian, et al 2017[10]	★	★	★	−	★	★	−	★	★	**7**
Fujihara, et al 2017[11]	★	★	★	★	-	★	★	★	-	**7**
Mirzaei, et al 2017[12]	★	★	★	★	★	★	★	★	★	**9**
Lee, et al 2018[13]	★	★	★	★	★	★	★	★	-	**8**
Li, et al 2018[14]	★	★	★	★	★	★	−	−	−	**6**
Xu, et al 2018[15]	★	★	★	★	★	★	★	★	★	**9**
Zhang, et al 2018[16]	★	★	★	★	★	★	★	★	−	**8**
Cho, et al 2019[17]	★	★	★	★	★	★	★	-	-	**7**
Li, et al 2019[18]	★	★	★	★	★	★	-	-	-	**6**
Izumida, et al 2019[19]	★	★	★	★	★	★	★	★	★	**9**

*Follow−up completed or less than 20% subjects lost for prospective cohort, clear flowchart with numbers of participants included and excluded for retrospective cohort

References:

1. Hosseinpanah, F.; Barzin, M.; Sheikholeslami, F.; Azizi, F. Effect of different obesity phenotypes on cardiovascular events in tehran lipid and glucose study (tlgs). Am J Cardiol 2011, 107, 412-416.
2. Choi, K.M.; Cho, H.J.; Choi, H.Y.; Yang, S.J.; Yoo, H.J.; Seo, J.A.; Kim, S.G.; Baik, S.H.; Choi, D.S.; Kim, N.H. Higher mortality in metabolically obese normal-weight people than in metabolically healthy obese subjects in elderly koreans. Clinical Endocrinology 2013, 79, 364-370.
3. Keihani, S.; Hosseinpanah, F.; Barzin, M.; Serahati, S.; Doustmohamadian, S.; Azizi, F. Abdominal obesity phenotypes and risk of cardiovascular disease in a decade of follow-up: The tehran lipid and glucose study. Atherosclerosis 2015, 238, 256-263.

4. Luo, D.; Liu, F.; Li, X.; Yin, D.; Lin, Z.; Liu, H.; Hou, X.; Wang, C.; Jia, W. Comparison of the effect of 'metabolically healthy but obese' and 'metabolically abnormal but not obese' phenotypes on development of diabetes and cardiovascular disease in chinese. Endocrine 2015, 49, 130-138.
5. Mirbolouk, M.; Asgari, S.; Sheikholeslami, F.; Mirbolouk, F.; Azizi, F.; Hadaegh, F. Different obesity phenotypes, and incident cardiovascular disease and mortality events in elderly iranians: Tehran lipid and glucose study. Geriatrics and Gerontology International 2015, 15, 449-456.
6. Sung, K.C.; Ryu, S.; Cheong, E.S.; Kim, B.S.; Kim, B.J.; Kim, Y.B.; Chung, P.W.; Wild, S.H.; Byrne, C.D. All-cause and cardiovascular mortality among koreans: Effects of obesity and metabolic health. American Journal of Preventive Medicine 2015, 49, 62-71.
7. Twig, G.; Gerstein, H.C.; Shor, D.B.A.; Derazne, E.; Tzur, D.; Afek, A.; Tirosh, A. Coronary artery disease risk among obese metabolically healthy young men. European Journal of Endocrinology 2015, 173, 305-312.
8. Kim, N.H.; Seo, J.A.; Cho, H.; Seo, J.H.; Yu, J.H.; Yoo, H.J.; Kim, S.G.; Choi, K.M.; Baik, S.H.; Choi, D.S., et al. Risk of the development of diabetes and cardiovascular disease in metabolically healthy obese people. Medicine (United States) 2016, 95.
9. Yang, H.K.; Han, K.; Kwon, H.S.; Park, Y.M.; Cho, J.H.; Yoon, K.H.; Kang, M.I.; Cha, B.Y.; Lee, S.H. Obesity, metabolic health, and mortality in adults: A nationwide population-based study in korea. Scientific reports 2016, 6, 30329.
10. Doustmohamadian, S.; Serahati, S.; Barzin, M.; Keihani, S.; Azizi, F.; Hosseinpanah, F. Risk of all-cause mortality in abdominal obesity phenotypes: Tehran lipid and glucose study. Nutrition, Metabolism and Cardiovascular Diseases 2017, 27, 241-248.
11. Fujihara, K.; Matsubayashi, Y.; Yamamoto, M.; Osawa, T.; Ishizawa, M.; Kaneko, M.; Matsunaga, S.; Kato, K.; Seida, H.; Yamanaka, N., et al. Impact of body mass index and metabolic phenotypes on coronary artery disease according to glucose tolerance status. Diabetes and Metabolism 2017, 43, 543-546.
12. Mirzaei, B.; Abdi, H.; Serahati, S.; Barzin, M.; Niroomand, M.; Azizi, F.; Hosseinpanah, F. Cardiovascular risk in different obesity phenotypes over a decade follow-up: Tehran lipid and glucose study. Atherosclerosis 2017, 258, 65-71.
13. Lee, H.J.; Choi, E.K.; Lee, S.H.; Kim, Y.J.; Han, K.D.; Oh, S. Risk of ischemic stroke in metabolically healthy obesity: A nationwide population-based study. PloS one 2018, 13, e0195210.
14. Li, L.; Chen, K.; Wang, A.P.; Gao, J.Q.; Zhao, K.; Wang, H.B.; Dou, J.T.; Lv, Z.H.; Wang, B.A.; Yan, W.H., et al. Cardiovascular disease outcomes in metabolically healthy obesity in communities of beijing cohort study. International Journal of Clinical Practice 2018.
15. Xu, Y.; Li, H.; Wang, A.; Su, Z.; Yang, G.; Luo, Y.; Tao, L.; Chen, S.; Wu, S.; Wang, Y., et al. Association between the metabolically healthy obese phenotype and the risk of myocardial infarction: Results from the kailuan study. Eur J Endocrinol 2018, 179, 343-352.
16. Zhang, R.; Dong, S.Y.; Wang, W.M.; Fei, S.Y.; Xiang, H.; Zeng, Q. Obesity, metabolic abnormalities, and mortality in older men. Journal of Geriatric Cardiology 2018, 15, 422-427.
17. Cho, Y.K.; Kang, Y.M.; Yoo, J.H.; Lee, J.; Park, J.Y.; Lee, W.J.; Kim, Y.J.; Jung, C.H. Implications of the dynamic nature of metabolic health status and

obesity on risk of incident cardiovascular events and mortality: A nationwide population-based cohort study. Metabolism 2019.
18. Li, H.; He, D.; Zheng, D.; Amsalu, E.; Wang, A.; Tao, L.; Guo, J.; Li, X.; Wang, W.; Guo, X. Metabolically healthy obese phenotype and risk of cardiovascular disease: Results from the china health and retirement longitudinal study. Archives of Gerontology and Geriatrics 2019, 82, 1-7.
19. Izumida, T.; Nakamura, Y.; Ishikawa, S. Impact of body mass index and metabolically unhealthy status on mortality in the japanese general population: The jms cohort study. PLoS One 2019, 14, e0224802.

Figure S1. Meta-regression bubble plot of the correlation between log odds ratio of cardiovascular disease and age.

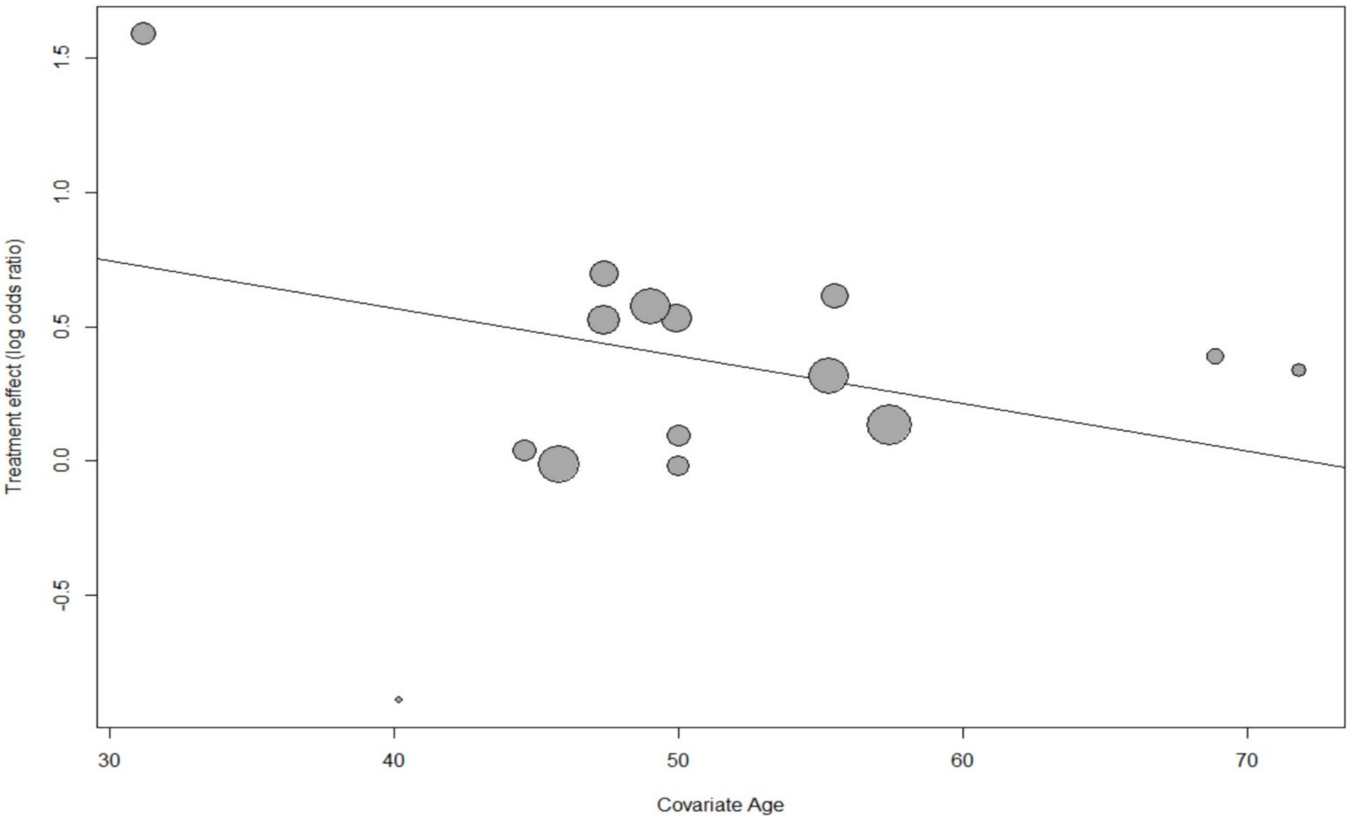

Each bubble represented a study and bubble size represented the sample size of the study. The regression line showed a nonsignificant trend for declining risk with age increased (*p*=0.20).

Figure S2. Meta-regression bubble plot of the correlation between log odds ratio of cardiovascular disease and the proportion of smoker.

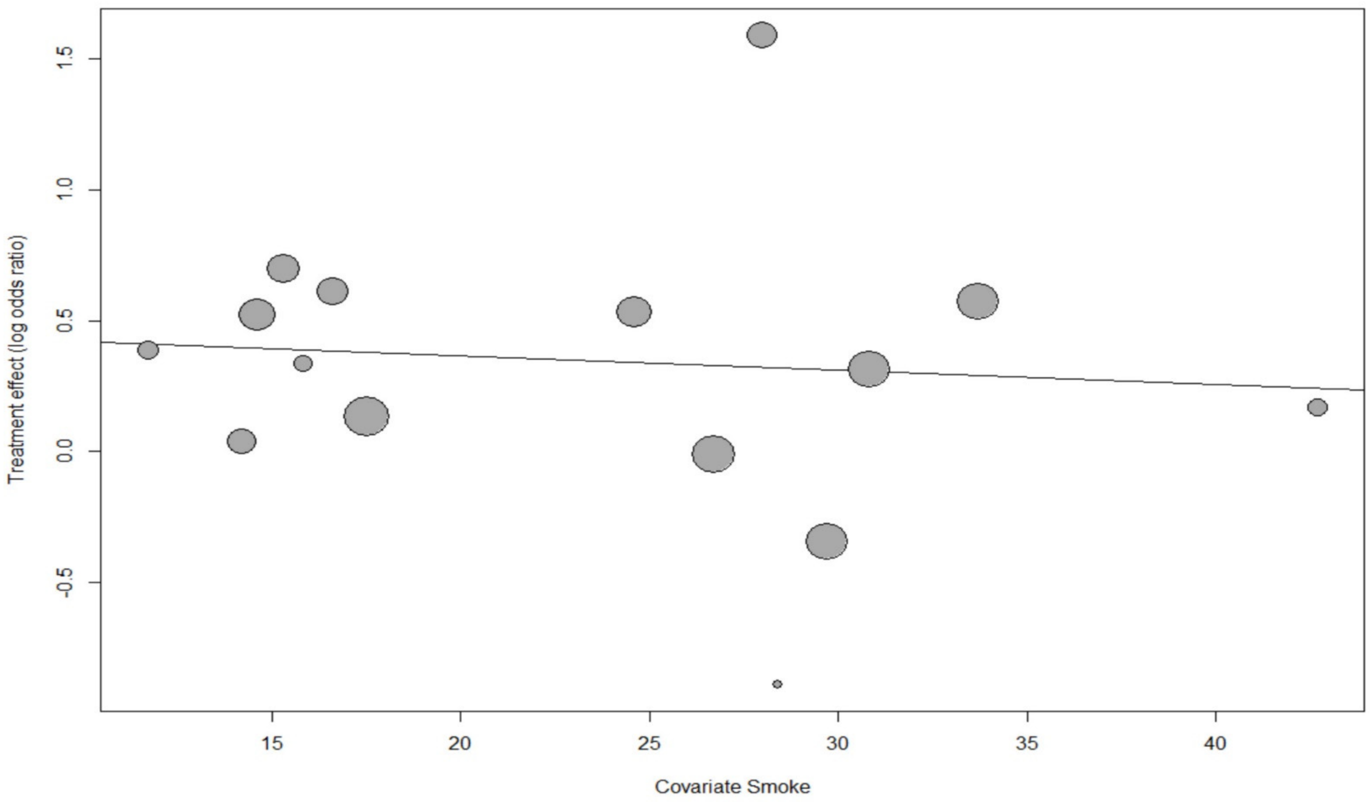

Each bubble represented a study and bubble size represented the sample size of the study. The regression line showed a nonsignificant trend for declining risk with smoker proportion increased (p=0.73).

Figure S3. Sensitivity analyses of metabolically healthy obesity and risk of cardiovascular disease by omitting each study.

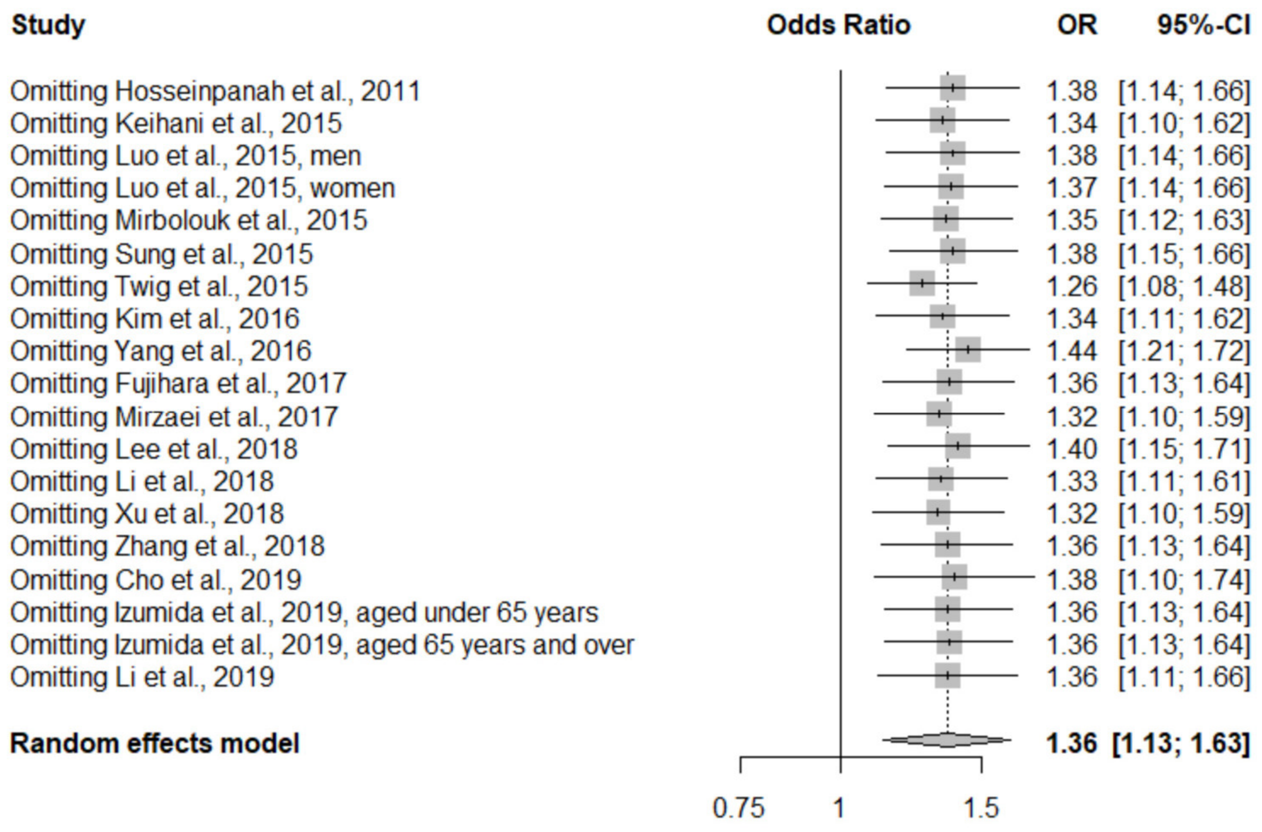

OR, odds ratio; CI, confidence interval

Table S5. Sensitivity analyses of participants with metabolically healthy obesity and risk of cardiovascular disease.

	Risk of cardiovascular disease		
	OR (95% CI)	I^2 (%)	Numbers of studies
Overall	1.36 (1.13−1.63)	75%	17
Articles with mean follow up duration at least 5 years	1.42 (1.04−1.94)	82%	12

OR, odds ratio; CI, confidence interval

Figure S4. The funnel plot standard error and odds ratio of cardiovascular disease showing study dispersion.

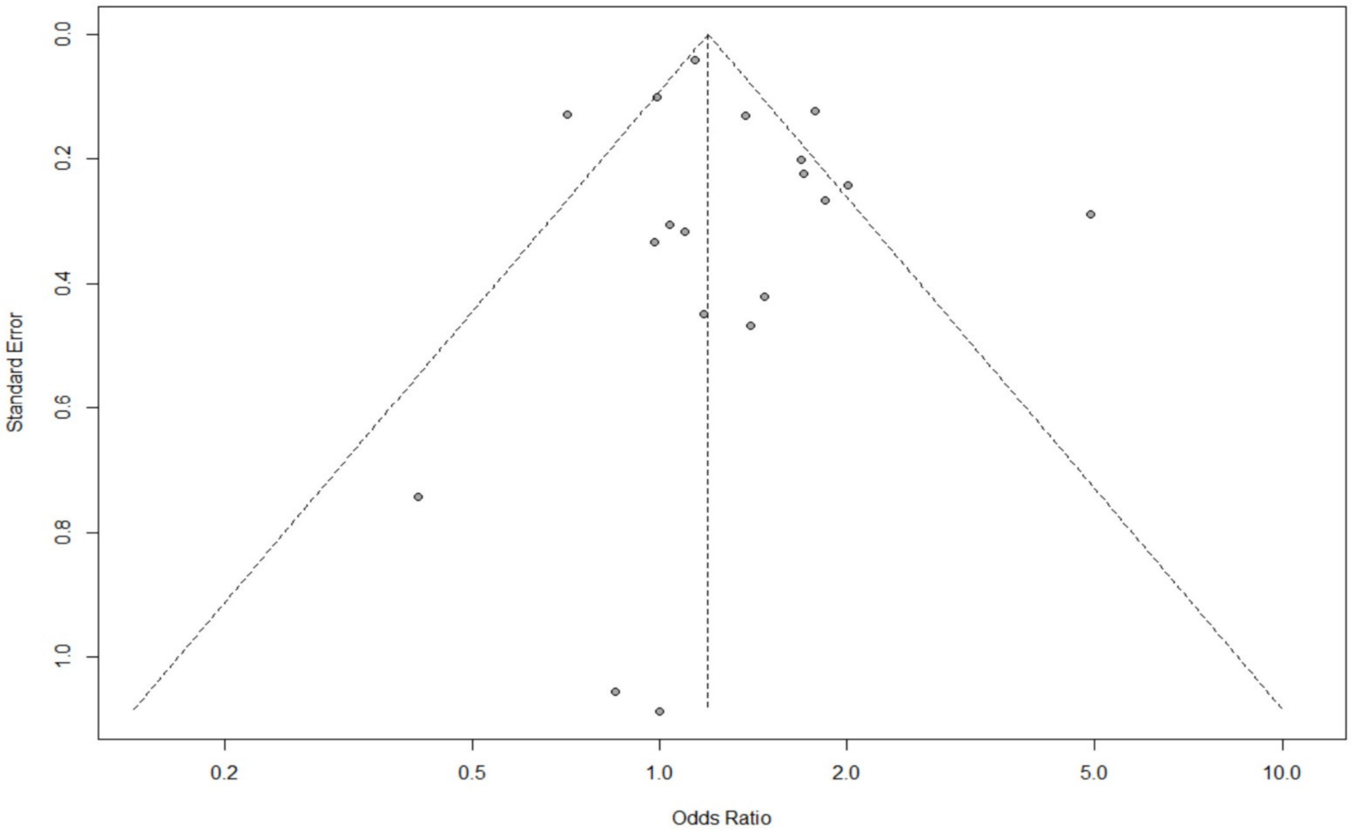

Egger’s test, slope = 0.11, $p = 0.29$

Figure S5. Forest plot of all-cause mortality, comparing participants with metabolically healthy obesity and participants with metabolically healthy non-obesity.

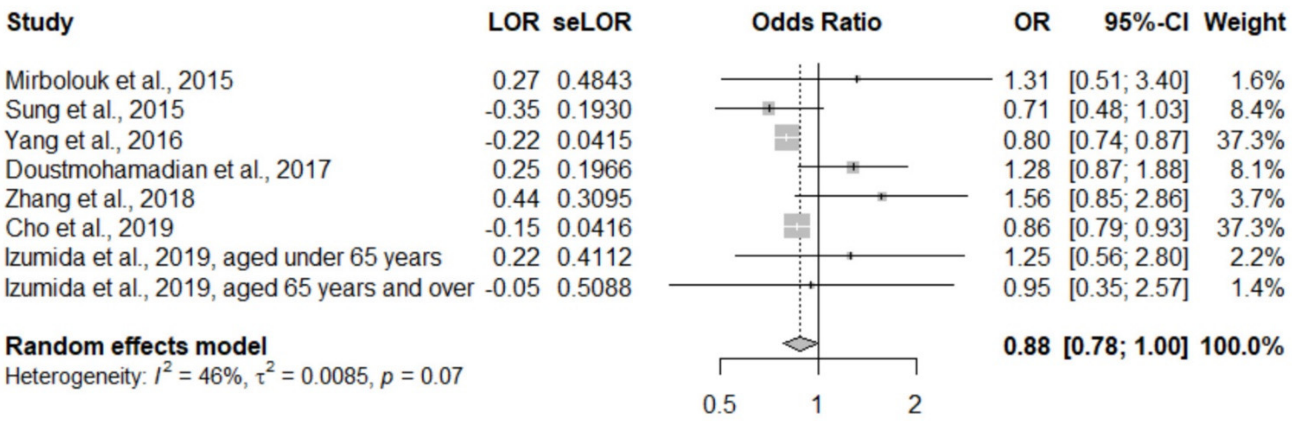

CI, confidence interval; LOR, logarithms of the odds ratio; OR, odds ratio; se, standard error

Figure S6. Sensitivity analyses of metabolically healthy obesity and risk of all-cause mortality by omitting each study.

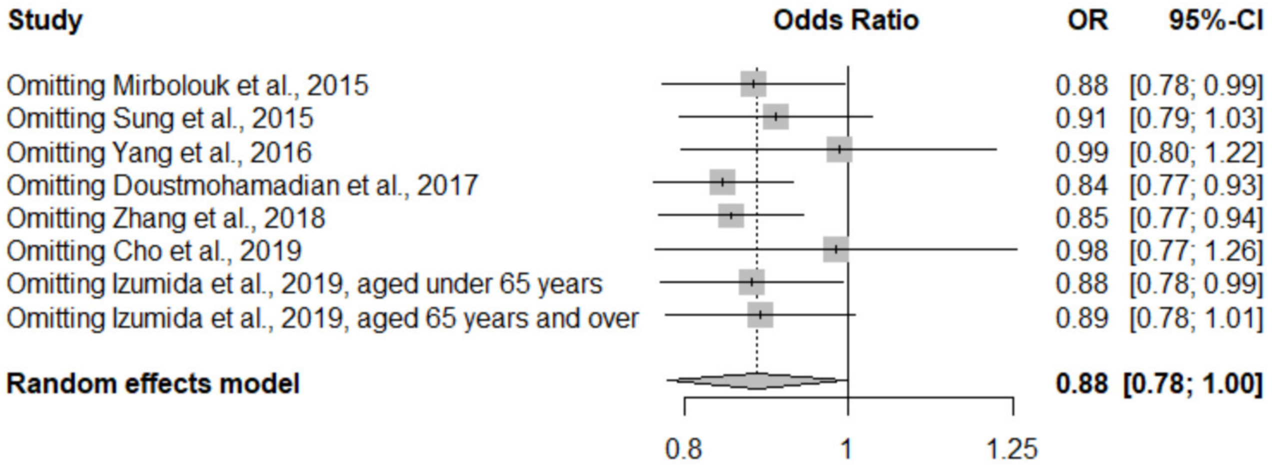

OR, odds ratio; CI, confidence interval

Table S6. Sensitivity analyses of participants with metabolically healthy obesity and risk of all-cause mortality.

	Risk of all-cause mortality		
	OR (95% CI)	I^2 (%)	Numbers of studies
Overall	0.88 (0.78−1.00)	46%	7
Articles with mean follow up duration at least 5 years	0.98 (0.77−1.26)	52%	6

OR, odds ratio; CI, confidence interval

Figure S7. The funnel plot standard error and odds ratio of all-cause mortality showing study dispersion.

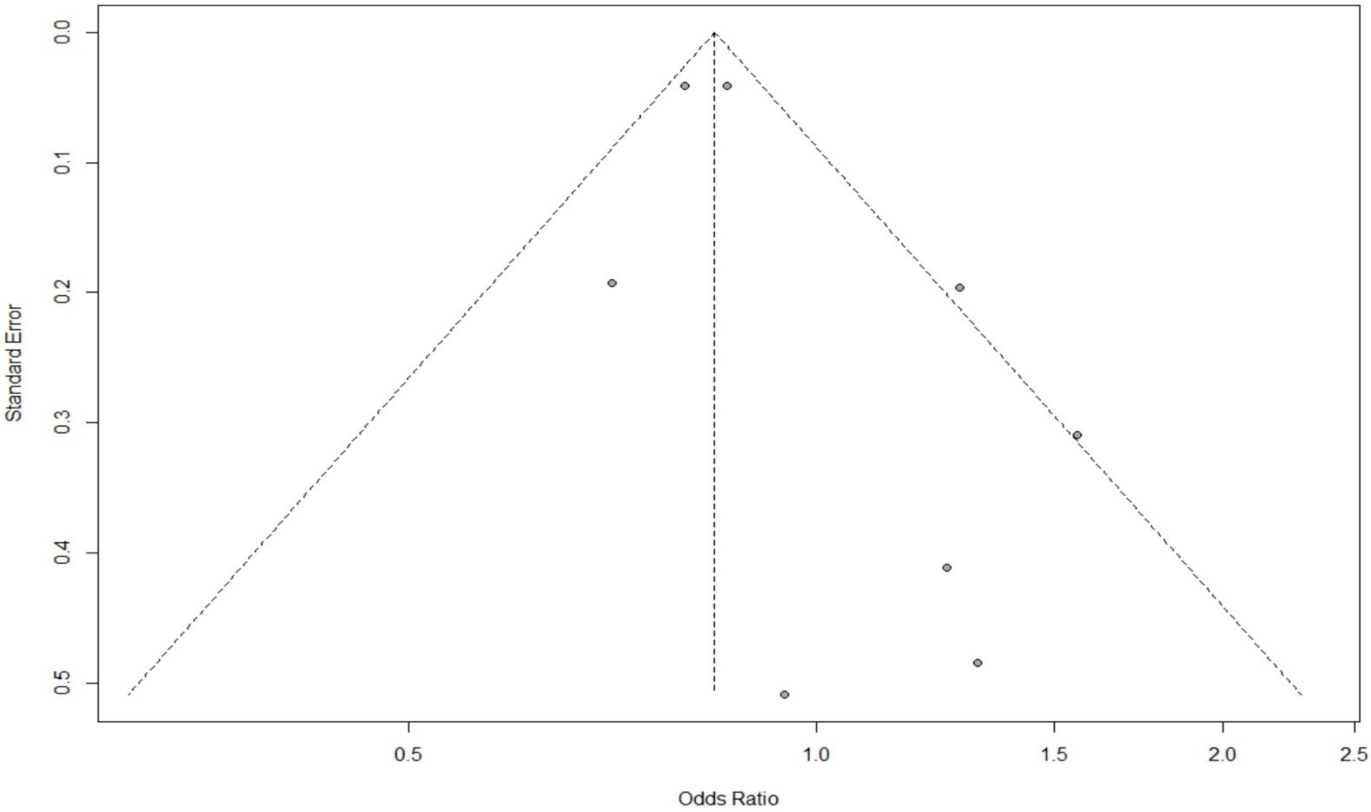

Egger's test, slope = –0.23, p = 0.10
